# [^11^C]phenytoin revisited: synthesis by [^11^C]CO carbonylation and first evaluation as a P-gp tracer in rats

**DOI:** 10.1186/2191-219X-2-36

**Published:** 2012-07-02

**Authors:** Joost Verbeek, Jonas Eriksson, Stina Syvänen, Maaike Labots, Elizabeth C M de Lange, Rob A Voskuyl, Martinus P J Mooijer, Marissa Rongen, Adriaan A Lammertsma, Albert D Windhorst

**Affiliations:** 1 Department of Nuclear Medicine & PET Research, Radionuclide Centre, VU University Medical Center, P.O. box 7057, Amsterdam 1081, HV, The Netherlands; 2 Division of Pharmacology, LACDR, Leiden University, Leiden, 2300, RA, The Netherlands; 3 Epilepsy Institutes of The Netherlands Foundation (SEIN), Heemstede, 2103, SW, The Netherlands

**Keywords:** Phenytoin, PET, Tariquidar, Probenecid, [^11^C]CO, BBB, P-gp, [^11^C]CO_2_ purification

## Abstract

**Background:**

At present, several positron emission tomography (PET) tracers are in use for imaging P-glycoprotein (P-gp) function in man. At baseline, substrate tracers such as *R*-[^11^C]verapamil display low brain concentrations with a distribution volume of around 1. [^11^C]phenytoin is supposed to be a weaker P-gp substrate, which may lead to higher brain concentrations at baseline. This could facilitate assessment of P-gp function when P-gp is upregulated. The purpose of this study was to synthesize [^11^C]phenytoin and to characterize its properties as a P-gp tracer.

**Methods:**

[^11^C]CO was used to synthesize [^11^C]phenytoin by rhodium-mediated carbonylation. Metabolism and, using PET, brain pharmacokinetics of [^11^C]phenytoin were studied in rats. Effects of P-gp function on [^11^C]phenytoin uptake were assessed using predosing with tariquidar.

**Results:**

[^11^C]phenytoin was synthesized via [^11^C]CO in an overall decay-corrected yield of 22 ± 4%. At 45 min after administration, 19% and 83% of radioactivity represented intact [^11^C]phenytoin in the plasma and brain, respectively. Compared with baseline, tariquidar predosing resulted in a 45% increase in the cerebral distribution volume of [^11^C]phenytoin.

**Conclusions:**

Using [^11^C]CO, the radiosynthesis of [^11^C]phenytoin could be improved. [^11^C]phenytoin appeared to be a rather weak P-gp substrate.

## Background

The blood–brain barrier (BBB) is a tightly connected cell layer that protects the brain from harmful substances and regulates transfer of various compounds between the brain and blood. The tight junctions between endothelial cells of the BBB force drugs to penetrate through rather than between these cells, either by passive diffusion, active influx or active efflux [1,2]. Active influx and efflux are processes that can transport molecules against a concentration gradient, hence requiring ATP-provided energy. P-glycoprotein (P-gp) is an ATP-dependent 170- to 180-kDa transmembrane glycoprotein present at the luminal side of the BBB where it prevents its substrates from entering the brain. This phenomenon may, however, also be associated with drug-resistance in several diseases, such as temporal lobe epilepsy [3-5], depression [6] and HIV [7]. In addition, a change in P-gp function at the BBB has been proposed as a possible link in the etiology of several neurological diseases [6-8].

A number of positron emission tomography (PET) tracers [9,10] for imaging P-gp function in man have been described, and they are all strong substrates of P-gp. Strong substrate tracers such as ^11^C]verapamil [11-13], ^11^C]desmethyl loperamide [14], ^11^C]laniquidar [15,16] and ^11^C]tariquidar [17] show a relatively low baseline uptake in both rat and human brain, with a brain-to-plasma equilibrium concentration ratio, often referred to as the volume of distribution (*V*_T_), of about 1.

Phenytoin, an anti-epileptic drug, is a relatively weak P-gp substrate. Therefore, in the present study, ^11^C]phenytoin was selected as a potential P-gp tracer. As a weak P-gp substrate and under normal physiological conditions, ^11^C]phenytoin is expected to have higher brain uptake than that of a strong substrate. When P-gp is upregulated, ^11^C]phenytoin might be a more sensitive tracer to detect changes in P-gp function than a strong substrate tracer. For the latter tracer, the already low signal will be reduced even further in case of P-gp overexpression, and consequently, this change in signal might be lost in noise. A dosage of 14 mg/kg ^11^C]phenytoin in rhesus monkeys resulted in a high brain uptake, which is consistent with phenytoin being a weak P-gp substrate [18]. Moreover, phenytoin is a registered drug, which facilitates easy translation from preclinical use to human PET applications.

Transition metal-mediated carbonylation using ^11^C]CO is a versatile method for the synthesis of compounds labeled with carbon-11 in the carbonyl position, e.g., amides, esters, ketones and carbamates with high specific activity [19-22]. Previously, it has been shown that rhodium-mediated reactions with ^11^C]CO, forming ureas, are rapid and provide high yield [23-25]. Therefore, in the present, study ^11^C]CO was used to synthesize ^11^C]phenytoin by rhodium-mediated carbonylation with the aim of improving both specific activity and radiochemical yield of ^11^C]phenytoin compared with an earlier method based on ^11^C]COCl_2_[26], which resulted in low specific activity of the product following a rather tedious radiochemistry [26,27]. The purpose of the present study was to synthesize ^11^C]phenytoin by carbonylation and to assess its characteristics as a P-gp substrate in rats *in vivo*.

## Methods

### General

Chemicals were obtained from Sigma Aldrich (St. Louis, MO, USA) and were used without further purification. All reactions were carried out under an argon atmosphere unless stated otherwise. [^11^C]CO_2_ was produced using an IBA Cyclone 18/9 cyclotron (IBA, Louvain-La-Neuve, Belgium) by the ^14^N(p,α)^11^C nuclear reaction performed in a 0.5% O_2_/N_2_ gas mixture. Water was purified over a Millipore filter (Millipore, Leiden, The Netherlands). The silica gel used as stationary phase for flash column chromatography was grade 9385, 230 to 400 mesh, 60 Å and obtained from Sigma Aldrich. Chemical shifts (*δ*) of nuclear magnetic resonance (NMR) spectra were defined relative to the signal of the solvent (7.26 for CDCl_3_ and 2.50 for DMSO-*d6*) and measured on a Bruker Avance 250 (Bruker, Billerica, MA, USA), with *δ* reported in parts per million relative to the solvent. Infrared (IR) spectra were obtained using a Shimadzu FTIR-8400 S (Shimadzu, Kyoto, Japan), and wavelengths (*ν*) are reported in per centimeter and (s), (m) and (w), indicating strong, moderate and weak signal, respectively. Electrospray ionization high-resolution mass spectrometry (ESI-HRMS) was carried out using a Bruker microTOF-Q instrument in positive ion mode (capillary potential of 4.500 V); electrospray ionization mass spectrometry was carried out using an AB Sciex Qtrap 5500 LC-MS/MS instrument (AB Sciex, Foster City, CA, USA) in negative ion mode. Semi-preparative high performance liquid chromatography (HPLC) was performed on an ACE 5-μM C18, 250*10-mm column (Alltech, Nicholasville, KY, USA) using the following settings: wavelength 230 nm, eluent MeCN/H_2_O/TFA 60/40/0.15 (*v*/*v*/*v*) and flow 4 mL·min^−1^ (method A). Analytical HPLC was performed on a Kromasil 100 C18 10-μM (4.6 to 250 mm) column (Grace Alltech, Breda, The Netherlands) with the following settings: wavelength 230 nm, eluent acetonitrile/water/diisopropylamide 75/25/0.2 (*v*/*v*/*v*) and flow 1 mL·min^−1^ (method B). Metabolite analysis was performed on a Gemini C18 5-μm (10 to 250 mm) column (Phenomenex, Torrance, CA, USA) (method C) with gradient and a mixture of (A) acetonitrile and (B) 0.1% trifluoro acetic acid in H_2_O as eluent according to the following scheme: 0 min, 90% B at 0.25 mL·min^−1^; 0.5 min, 20% B at 4 mL·min^−1^; 9.0 min, 30% B at 4 mL·min^−1^; 13 min, 30% B at 4 mL·min^−1^; 14.0 min, 90% B at 4.0 mL·min^−1^ and 15 min, 90% B at 0.25 mL·min^−1^. Tetrahydrofuran (THF), which was used during experiments, was distilled over LiAlH_4_ just prior to the experiments unless stated otherwise.

### Synthesis

#### *Synthesis of azido-diphenyl-acetic acid (2)*

Sodium azide 1.78 g (27.5 mmol) was dissolved in a mixture of water (4.5 mL) and CH_2_Cl_2_ (7.5 mL). After cooling the solution in an ice bath, trifluoromethanesulfonic anhydride (0.93 mL, 5.6 mmol) was added slowly for 5 min and then stirred for 2 h at 4°C to form triflyl azide [23]. The dichloromethane layer was separated, and the aqueous layer was extracted twice with 4 mL of CH_2_Cl_2_. The combined organic layers were washed with a saturated aqueous Na_2_CO_3_ solution (10 mL). The resulting solution of triflyl azide in CH_2_Cl_2_ was used without further purification and added to a mixture of aminodiphenylacetic acid 1 (0.65 g, 2.8 mmol), K_2_CO_3_ (0.58 g, 4.2 mmol) and Cu(II)SO_4_·5H_2_O (7.0 mg, 28 μmol) in a mixture of H_2_O (9 mL) and CH_3_OH (18 mL). Subsequently, this mixture was stirred overnight at room temperature. After evaporation of CH_2_Cl_2_ and CH_3_OH under reduced pressure, the residue was washed twice with ethyl acetate (10 mL) in a separation funnel. The water layer was acidified to pH 2 with concentrated HCl and subsequently extracted four times with CH_2_Cl_2_ (10 mL). The combined organic layers were dried over Na_2_SO_4_, and the solvent was evaporated under reduced pressure. The residual oil was purified by flash column chromatography with a mixture of CH_2_Cl_2_/CH_3_OH 97/3 (*v*/*v*). The fractions containing the product were collected, and the solvent was evaporated under reduced pressure to obtain 2 (0.53 g, 2.1 mmol) as a white solid. Yield 75%, retardation factor (Rf) of 0.15 (CH_2_Cl_2_/CH_3_OH 97/3 (*v*/*v*)). ^1^H-NMR (CDCl_3_) δ: 7.28 to 7.39 (10 H, m, aromatic protons). ^13^C-NMR (CDCl_3_) *δ*: 128.14, 128.52, 128.88, 138.09. Infrared spectrum: 2,112 cm^−1^ (s, N_3_) and 1,744 cm^−1^ (s, C = O).

#### *Synthesis of azidodiphenylacetamide (3)*

Azidodiphenylacetic acid 2 (532 mg, 2.1 mmol) was dissolved in dry acetonitrile (20 mL) and SOCl_2_ (5 mL). The resulting solution was refluxed for 1.5 h to form azidodiphenylacetamide 3. Acetonitrile and SOCl_2_ were then removed under reduced pressure, which was followed by addition of dry toluene (10 mL) and removal of residual SOCl_2_ by co-evaporation under reduced pressure. The co-evaporation procedure with toluene was then repeated once more. The remaining solid was dissolved in dry acetonitrile (20 mL), and ammonia gas was gently bubbled trough the solution for 30 min; after which, the flask was closed and the mixture was stirred overnight at room temperature. After the solvent was evaporated under reduced pressure, the residue was dissolved in CH_2_Cl_2_ (30 mL), and this solution was washed twice with water (20 mL). The CH_2_Cl_2_ solution was dried over Na_2_SO_4_ and filtered, and the filtrate was evaporated under reduced pressure. The crude product was purified by flash column chromatography with ethyl acetate/hexane 45/55 (*v*/*v*). The fractions containing the product were collected, and the solvent was evaporated under reduced pressure to obtain 402 mg (1.6 mmol) of 3 as a white solid. Yield 76%, Rf 0.20 (ethyl acetate/hexane 45/55 (*v*/*v*)) ^1^H-NMR (CDCl_3_) *δ*: 6.09 (1H, s, CONH_2_), 6.55 (1H, s, CONH_2_), 7.39 to 7.52 (10H, m, aromatic protons). ^13^C-NMR (CDCl_3_) 128.33, 128.57, 128.74, 138.75, 172.69. HRMS (ESI) *m/z* calculated for C_14_H_12_N_4_O [M^+^ Na^+^] 275.0893 found 275.0903. Elemental analysis calculated: C 66.65, H 4.79, N 22.21; found: C 66.65, H 4.82, N 22.15. Infrared spectrum: 3,452 cm^−1^ (m, C = ONH_2_), 2,110 cm^−1^ (s, N_3_) and 1,682 cm^−1^ (s, C = ONH_2_).

### Synthesis of [^11^C]phenytoin

A solution of rhodium(II) acetate dimer (0.35 mg, 0.80 μmol), 1,2-bis(diphenylphosphino)ethane (0.90 mg, 2.2 μmol) and 3 (4.3 mg, 17 μmol) in freshly distilled THF (400 μL) was prepared in a septum-equipped vial (2.0 mL). The vial was gently heated until there was a color change from light yellow to dark orange, which indicated that the desired catalytic complex was formed.

**Figure 1:**
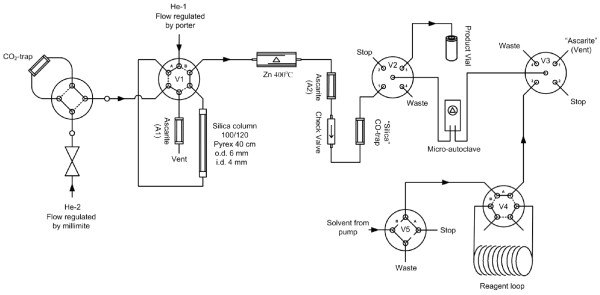
**Schematic overview of the synthesis unit used to synthesize [**^**11**^**C]CO.**

The synthesis of ^11^C]phenytoin was performed using a semiautomatic synthesis module (Figure 1). The module was built in-house based on the technology developed for ^11^C]CO carbonylation [28]. ^11^C]CO_2_ was first trapped and concentrated on silica gel immersed in liquid nitrogen (−196°C). Valve V1 was then switched to confine the ^11^C]CO_2_ before heating the trap to room temperature. Next, the valve was switched again to release the concentrated ^11^C]CO_2_ into a stream of helium (20 mL/min) and over a gas purification column (silica gel 100/120 mesh (Alltech), 5 mass% water). Subsequently, the ^11^C]CO_2_ was reduced over zinc at 400°C. Formed ^11^C]CO was trapped on a silica trap immersed in liquid nitrogen, and unreacted ^11^C]CO_2_ was trapped on an ascarite column (A2).

Valve V2 was switched to position 2 before the CO trap was heated to ambient temperature. This was followed by the transfer of [^11^C]CO to the micro-autoclave in a stream of helium (3 bar) by switching valve V2 to position 1. After this transfer, valve V2 was switched back to position 2. The freshly prepared precursor solution was loaded on the reagent loop and transferred to the micro-autoclave using THF pumped at a pressure of up to 300 bar. This high pressure forced both [^11^C]CO and the helium transfer gas to dissolve in the reagent solution in the micro-autoclave, which then was heated for 5 min at 120°C to facilitate the carbonylation reaction. Next, the reaction mixture was transferred to a vacuumized vial by switching valve V2 to position 3. The product solution was degassed with helium (10 mL) to remove residual [^11^C]CO and other gaseous compounds. Next, the reaction mixture was diluted with H_2_O (1 mL), injected on the preparative HPLC system and purified using HPLC method A. [^11^C]phenytoin, with a retention time in the range of 7 to 9 min, was collected directly into a vortex evaporator (PET Evap, Scansys Laboratortechnik, Copenhagen, Denmark). During fraction collection, the HPLC eluent was evaporated continuously at 60°C under N_2_ gas at high flow. After the HPLC eluent was removed, buffered 0.9% NaCl aqueous solution (12 mL, pH 7,4; 8.4-mM Na_2_HPO_4_/1.8-mM NaH_2_PO_4_) was added, and the reformulated [^11^C]phenytoin was passed over a Millex GV filter (0.22 μM, Millipore, Billerica, MA, USA) and dispensed into a sterile product vial. The chemical and radiochemical purity was assessed using analytical HPLC method B. All radiochemical yields reported are based on the amount of initial radioactivity at the start of the carbonylation reaction, as determined by measuring the reaction mixture after transfer from the micro-autoclave. The radioactive residues remaining in the micro-autoclave after transfer were negligible.

### ^11^C/^13^C co-labeling of phenytoin

A solution of 3 (10 mg, 40 μmol), rhodium(II) acetate dimer (0.7 mg, 1.6 μmol) and 1,2-bis(diphenylphosphino)ethane (DPPE; 1.8 mg, 4.5 μmol) in THF (300 μL) prepared as in the previous section was loaded on a 1-mL injection loop, which was preloaded with [^13^C]CO gas. The content of the loop was transferred to the autoclave containing [^11^C]CO as described in the previous section. The autoclave was heated for 20 min at 120°C. Next, the reaction mixture was purified using HPLC method A. The collected fraction was analyzed using HPLC method B to assess identity and radiochemical and chemical purities. After decay, acetonitrile was removed from a rotary evaporator, after which the water fraction was extracted with dichloromethane (4 × 10 mL). The combined portions of dichloromethane were dried over Na_2_SO_4_ and filtered. Rotary evaporation yielded 1.3 mg (13% yield) of [^13^C]phenytoin. Analysis with ^13^C-NMR (DMSO-d6) gave a spectrum with signals at 156.1, 128.5 and 126.6 ppm. These signals were also present in the spectrum taken with isotopically unmodified phenytoin. The signal at 156.1 ppm had the highest intensity and corresponded to the quaternary carbon of phenytoin. This identity was also confirmed using an LC/MS/MS system with a negative ion peak at *m/z* 252.1.

### Animals

Adult male Sprague–Dawley rats (Harlan, Horst, The Netherlands) weighing 200 to 224 g at arrival were housed in groups of five to six per cage with unrestricted access to food (Teklad Global 16% Protein Rodent Diet, Harlan, Madison, WI, USA) and water. Rats were kept at a constant temperature of 21°C and in a 12-h light/dark cycle in which lights were switched on at 8:00 am. Animal procedures were performed in accordance with the Dutch laws on animal experimentation.

Approximately 1 week after habituation, rats were used in either PET or metabolite studies. Rats were anesthetized via a nose mask with 4% and 2% isoflurane in oxygen for induction and maintenance, respectively, at a rate of 1 L·min^−1^. The femoral vein and artery were cannulated 1 to 2 h prior to each study of [^11^C]phenytoin and tariquidar administration, and blood sampling, respectively. Blood oxygen saturation and body temperature were monitored throughout surgery and experiments. Heating and oxygen supply were adjusted to maintain a body temperature > 36°C and an oxygen saturation > 96%.

### Metabolite analysis in the plasma and brain tissue

To study the metabolic profile of [^11^C]phenytoin in the plasma and brain, five male Sprague–Dawley rats were injected with 150 to 250 MBq [^11^C]phenytoin in the tail vein under isoflurane anesthesia. Three of these animals were pretreated with 15 mg·kg^−1^ tariquidar 20 min prior to [^11^C]phenytoin administration. Three arterial blood samples of 0.5, 0.8 and 1.2 mL were withdrawn at 10, 30 and 45 min or at 5, 15 and 25 min after [^11^C]phenytoin injection, respectively. Immediately following the last blood sample, the rats were euthanized, and the brain was isolated. All blood samples were collected in heparin tubes and centrifuged at 4.000 rpm for 5 min at 4°C (Hettich Universal 16, Depex B.V., The Netherlands). Plasma was separated from blood cells and, between 0.3 and 0.7 mL, was loaded onto a tC-18 Sep-Pak (Waters, The Netherlands) and washed with 20 mL of water. The eluate was defined as the polar radiolabeled metabolite fraction. Thereafter, the tC-18 Sep-Pak was eluted with 1.5 ml of methanol. This eluate was defined as the nonpolar fraction and was analyzed using HPLC method C. Brain tissue was homogenized with an ultrasonic homogenizer (Braunsonic 1510, Kronberg, Germany) in water, under ice cooling, and subsequently centrifuged at 4,000 rpm for 5 min at 4°C. Separated supernatants were loaded onto a tC-18 Sep-Pak and washed with 20 mL water to obtain the polar fraction. The nonpolar fraction was eluted in 1.5 ml of methanol and analyzed with HPLC method C. The recovery of the metabolite analysis was validated to be above 95%.

### PET studies

Following cannulation of the femoral vein, animals were positioned in pairs in a double LSO/LYSO layer high-resolution research tomograph (CTI/Siemens, Knoxville, TN, USA) [29]. First, for attenuation and scatter correction purposes, a transmission scan was acquired using a 740-MBq two-dimensional fan-collimated ^137^Cs (662 keV) moving point source [30]. Next, a dynamic emission scan was acquired immediately following administration of 17.1 ± 2.1 MBq (mean ± SD) ^11^C]phenytoin to each rat. Two animals had one single ^11^C]phenytoin scan; two animals had two consecutive scans, and two had three consecutive scans. Finally, all animals underwent an ^18^F]FDG scan before being euthanized. An overview of acquired scans is listed in Table 1. Tariquidar, 15 mg·kg^−1^, was used to completely block P-gp, while probenecid, 150 mg·kg^−1^, was used to inhibit multi-resistant protein (MRP) efflux proteins. Tariquidar and probenecid were administered i.v. 20 min prior to the scan start as a 10-min infusion. All animals, except one, were scanned without any treatment prior to the first ^11^C]phenytoin scan. Of the animals that had two scans, one underwent two post-tariquidar scans (animal 4, Table 1). The aim of this particular set of scans was to ensure that the order of scans did not affect results. Animals that underwent three consecutive scans had one baseline, one post probenecid and one post-tariquidar scan. During each scan, emission data were acquired for 60 min in list mode and rebinned into the following frame sequence: 7 × 10, 1 × 20, 3 × 30, 2 × 60, 2 × 150, 2 × 300 and 4 × 600 s. Following corrections for decay, dead time, attenuation, random and scatter, scans were reconstructed using a 3D ordinary Poisson ordered subsets expectation maximization algorithm. This resulted in images with an average spatial resolution of 3 mm full width half maximum [29]. PET image data were analyzed using the freely available software package Amide 0.9 [31]. A magnetic resonance (MR)-based rat brain atlas [32] was used to define five regions of interest (ROIs): the hippocampus, occipital cortex, parietal cortex, caudate putamen and cerebellum. The MR atlas including all ROIs was aligned visually with the summed ^18^F]FDG image for each rat. ROIs were then projected onto all frames of all ^11^C]phenytoin scans, resulting in ^11^C]phenytoin time-activity curves for each scan and rat.

**Table 1:** **Overview of [**^**11**^**C]phenytoin scans**

**Animal**	**Scan 1**	**Scan 2**	**Scan 3**
1	Baseline	-	-
2	Baseline	-	-
3	Baseline	Tariquidar 15 mg·kg^−1^	-
4	Tariquidar 15 mg·kg^−1^	Tariquidar 15 mg·kg^−1^	-
5	Baseline	Probenecid 150 mg·kg^−1^	Tariquidar 15 mg·kg^−1^
6	Baseline	Probenecid 150 mg·kg^−1^	Tariquidar 15 mg·kg^−1^

During each [^11^C]phenytoin scan, arterial blood samples of 0.1 mL were taken at 20 and 40 s and at 1, 2, 5, 15, 30 and 60 min after [^11^C]phenytoin injection. For animals that underwent three scans, the amount of blood withdrawn was reduced by replacing the two samples at 15 and 30 min with a single 20-min sample. Plasma was obtained by centrifugation at 4,000 rpm for 5 min at 4°C, and activity in both plasma and whole blood was measured in an automated 1282 Compugamma CS Universal Gamma Counter (LKB Wallac, Turku, Finland), cross-calibrated against the PET scanner. Standardized uptake values (SUVs) as a function of time were derived by normalizing measured radioactivity concentrations against injected dose and body weight.

To obtain a model independent estimate of the brain-to-plasma partition coefficient, often referred to as volume of distribution, Logan graphical analysis [33] was used. Before Logan analysis, both brain and plasma concentrations were corrected for labeled metabolites. As metabolite fractions were not measured at each plasma or mid-frame time point, linear regression was used to estimate the fraction of intact ^11^C]phenytoin at each time point.

## Results and discussion

### Synthesis of phenytoin precursor

**Scheme 1:**
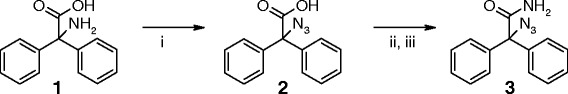
**The synthesis of azidodiphenylacetamide 3.** (i) Triflyl azide, K_2_CO_3_, Cu(II)SO_4_·5H_2_O, CH_2_Cl_2_, CH_3_OH, 16 hr, room temperature (RT), 75% yield. (ii) SOCl_2_, CH_3_CN, 90 min, reflux. (iii) NH_4_, CH_3_CN, 16 hr, RT, 76% yield.

The synthesis of azidodiphenylacetamide 3, as described previously by Hohenlohe-Oehringen [34], started from 2-bromo-2,2-diphenylacetonitrile followed by an azidation reaction to yield the crucial intermediate 2-azido-2,2-diphenylacetonitrile. However, in our hands, this intermediate could not be synthesized. Attempts to obtain 3 via the corresponding methylester or by first introducing an amide function were also unsuccessful as the subsequent conversion to the azide failed. Finally, 3 was synthesized in three reaction steps starting from aminodiphenylacetic acid 1 and diazo-transfer from triflyl azide [35] to form azidodiphenylacetic acid 2. Next, the amidation was performed by conversion to 2-azido-2,2-diphenylacetyl chloride, and subsequent treatment with ammonia gave azidodiphenylacetamide 3 in an overall yield of 57% after purification by flash column chromatography, which is shown in Scheme 1.

### Production of [^11^C]CO

**Figure 2:**
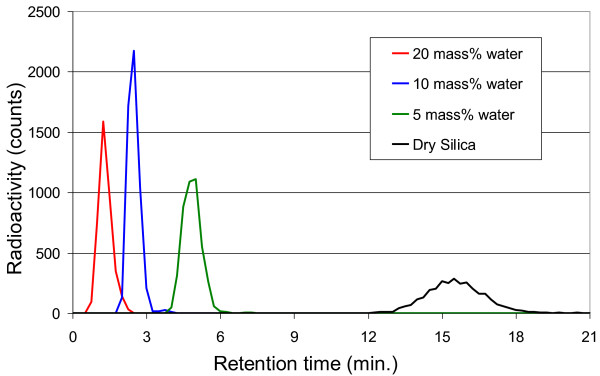
**Retention time of [**^**11**^**C]CO**_**2**_**on silica gel with varying mass percentages of water.** The silica gel was prepared by drying for 16 h at 120°C, after which water was added.

^11^C]phenytoin was synthesized by carbonylation of 3 using ^11^C]CO. Although it has been reported that heated zinc can reduce ^11^C]CO_2_ to ^11^C]CO, this reaction did not work in our lab [36]. A possible explanation could be the use of 0.5% oxygen in the target gas, which is a factor 10 higher than used previously [24,36]. To circumvent potential deactivation of zinc by rapid oxidation, a column containing silica gel (100/120 mesh) was implemented to chromatographically purify ^11^C]CO_2_. Retention of ^11^C]CO_2_ strongly depended on the mass percentage of water in the silica gel with less water resulting in a longer retention time, as depicted in Figure 2. On the other hand, molecular oxygen has little retention on silica gel, enabling the removal of O_2_ by discarding the effluent prior to the ^11^C]CO_2_ fraction.

By testing silica gel with different moisture contents, it was verified that the reduction proceeded reliably when ^11^C]CO_2_ eluted from the column between 4 to 9 min, while shorter or longer retention times resulted in permanent deactivation of the zinc. The optimum water content in the silica gel was determined to be 5 mass% of water. These observations did not support the assumption that O_2_ was the direct cause of zinc deactivation. A second possibility could be that the high O_2_ concentration in the target gas led to formation of nitrogen oxides during irradiation [37,38], which in turn caused the deactivation of zinc [39]. Among all nitrogen oxides, N_2_O is the most likely candidate to cause zinc deactivation based in part on its oxidizing properties. In addition, it is difficult to remove as it has similar chromatographic behavior as CO_2_ on silica gel, including peak broadening and increasing retention time with decreasing water content in the silica gel, as reported by Gamo et al. [40]. While the exact reason for the deactivation of zinc needs to be clarified in future studies, the silica gel column purification facilitated practical use of zinc in the ^11^C]CO synthesis. Typically, the zinc column needed to be replenished after seven to ten syntheses.

### Synthesis of [^11^C]phenytoin

The synthesis of ^11^C]phenytoin was performed in a high-pressure autoclave according to the general method developed by Kihlberg et al. [28]. Use of the high-pressure method provided an efficient way of transferring the minute amount of ^11^C]CO into the reaction solution and to circumvent low incorporation yields associated with poor solubility of ^11^C]CO.

**Figure 3:**
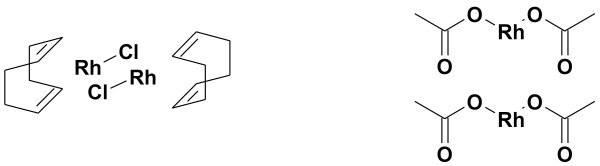
**Rhodium sources used: (Rh(cod)**_**2**_**) and rhodium(II) acetate dimer.**

Several rhodium catalyst complexes were explored during optimization of the [^11^C]phenytoin synthesis. The commercially available Wilkinson’s catalyst gave [^11^C]phenytoin in low yields, up to 4%, and generally poor conversion of [^11^C]CO to nonvolatile products. Next, the catalytic activity of complexes formed from chloro(1,5-cyclooctadiene)rhodium(I) dimer (Rh(cod)_2_) and with rhodium(II) acetate dimer, which are depicted in Figure 3, was explored.

**Figure 4:**
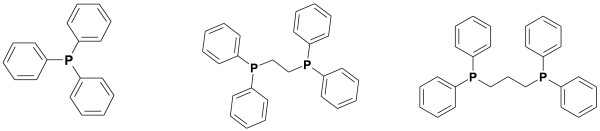
Phosphine ligands used: triphenylphosphine, DPPE and DPPP.

These rhodium sources were utilized in combination with three different phosphine ligands (Figure 4): triphenylphosphine, 1,2-bis(diphenylphosphino)ethane and 1,3-bis(diphenylphosphino)propane (DPPP). DPPE gave the best results of these three phosphines when combined with Rh(cod)_2_, with yields up to 22% and an average radiochemical yield of 11 ± 10%, but with poor reproducibility. Both DPPP and triphenylphosphine gave lower yields.

Next, rhodium(II) acetate dimer was investigated, and again, DPPE gave better results than DPPP and triphenylphosphine. In addition, yields proved to be more reproducible compared with Rh(cod)_2_. Preheating the reaction mixture improved both yield and reproducibility. The optimal amount of precursor 3 seemed to be around 17 μmol. A decrease in this amount led to a decrease in yield, while an increase did not improve the yield (Table 2).

**Table 2:** Yield as function of amount of precursor (3) for constant amount of rhodium acetate

**Precursor 3**	**Yield**
**(μmol)**	**(%)**
8.4	3
9.1	5
12	7
17	21
21	19

It should be noted that 200 μL out of the 400-μL precursor solution will be added to the micro-autoclave.

By varying the ratio of rhodium acetate dimer to DPPE, the optimum was found to be between 1.2 and 1.6 (Table 3). In addition, the effect of catalyst load was investigated. The optimum was obtained for 0.7 to 0.9 μmol rhodium acetate dimer and 1.7 to 2.3 μmol DPPE.

**Table 3:** Effect of rhodium acetate, DPPE and their ratio on yield

**Entry**	**Rhodium acetate**	**DPPE**	**Ratio**	**Yield**
	**(μmol)**	**(μmol)**	**DPPE/rhodium**	**(%)**
1	2.8	1.3	0.5	2
2	2.2	1.5	0.7	3
3	6.2	5.5	0.9	1
4	2.0	1.8	0.9	7
5	1.4	1.5	1.1	9
6	2.4	2.6	1.1	9
7	1.8	2.0	1.1	18
8	1.4	1.7	1.2	27
9	1.4	1.7	1.2	24
10	1.6	2.0	1.3	25
11	4.2	4.4	1.3	12
12	0.6	0.9	1.5	4
13	1.4	2.3	1.6	21
14	2.4	6.2	2.6	3

It should be noted that 200 μL out of the 400-μL precursor solution will be added to the micro-autoclave.

A reaction time of 5 min was deemed sufficient as longer times did not increase the yield. The reaction performed best at 120°C. No improvement was seen for higher temperatures, and the yield dropped to 4% at 80°C and to 9% at 100°C. Dimethylformamide was also attempted as a solvent but gave no yield.

**Scheme 2:**
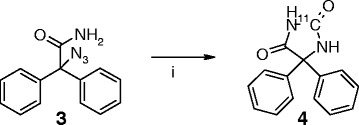
**Radiolabeling of [**^**11**^**C]phenytoin 4.** (i) [^11^C]CO, Rh(II) acetate dimer, DPPE, THF, 5 min, 250 bar, 120°C, 22 ± 4% overall yield.

After these optimization steps, rhodium acetate dimer in combination with DPPE gave reliable yields of 19% to 27%, as illustrated in Scheme 2. The (optimal) reaction conditions used were 16 to 18 μmol 3, 0.7 to 0.9 μmol rhodium acetate dimer and 1.7 to 2.3 μmol DPPE, in 400 μmol THF at 120°C for 5 min under a pressure of 250 bar for 5 min to yield 22 ± 4% decay-corrected (1 to 2 GBq) [^11^C]phenytoin with a specific activity of 277 ± 67 GBq μmol^−1^ at end of synthesis (EOS). It should be noted that via the loop, in practice, 200 μL out of the 400-μL precursor solution was added to the micro-autoclave.

**Figure 5:**
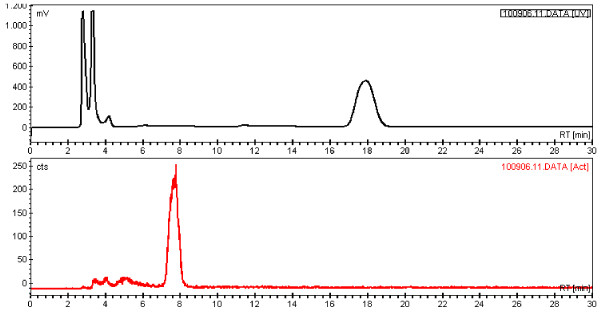
Typical preparative HPLC chromatogram with UV absorption (upper panel) and radioactivity (lower panel) signals.

Purification of [^11^C]phenytoin was performed by preparative HPLC using method A. The retention times of [^11^C]phenytoin and precursor 3 were approximately 8 and 18 min, respectively, as shown in Figure 5.

The standard method for removing the HPLC eluent is solid-phase extraction with commercially available cartridges. Several cartridges were tested: the Waters C18 plus, tC-18, C-18 light and C8, the ion exchange columns PS-OH and PS-HCO_3_ from Machery-Nagel (Düren, Germany), the Alltech SAX and the OASIS HLB (Waters Corp., Milford, MA, USA). Except for the PS-HCO_3_, however, all these cartridges suffered from either poor trapping, poor recovery or low reproducibility. Although the PS-HCO_3_ cartridge showed good trapping and good elution with acidic ethanol, an impurity, however, co-eluted with [^11^C]phenytoin, making this cartridge unsuitable for radiopharmaceutical production. As solid-phase extraction proved to be troublesome, the HPLC solvent was removed by means of a vortex evaporator (PET Evap, Scansys Laboratortechnik). The HPLC fraction containing [^11^C]phenytoin was transferred into the PET Evap, which was heated to 60°C. Evaporation was facilitated by a high flow of N_2_ gas. When collection of HPLC fractions was complete, the transfer line to the PET Evap was rinsed with 5 mL of water to recover the remaining [^11^C]phenytoin. Two minutes after the HPLC fraction was collected, evaporation was complete. Heating of the PET Evap was switched off; the N_2_ flow was reduced, and the product was washed out of the PET Evap with 10 mL PBS buffer in saline. After passing over a sterile filter (Millex GV, Millipore), the final product ready for injection was obtained in a yield of 22 ± 4%, decay-corrected (1 to 2 GBq), and a specific activity of 277 ± 67 GBq·μmol^−1^ at EOS with a total synthesis time of approximately 35 min. The radiochemical purity was ≥98%, and no visible UV peaks were observed in the analytical HPLC chromatogram.

The identity of [^11^C]phenytoin was further confirmed by co-synthesis of phenytoin with both [^13^C]CO and [^11^C]CO. Carbon NMR showed a high signal at 156.1 ppm, which corresponds to the carbonyl peak in the reference phenytoin. Next, the identity of [^13^C]phenytoin was also confirmed with mass spectrometry which, as expected, showed a negative ion peak at *m/z* 252.1. In addition, analytical HPLC confirmed that [^11^C]phenytoin was formed.

### Metabolite analysis

Metabolism of [^11^C]phenytoin in the plasma was relatively fast. Forty-five minutes after administration, 19% of radioactivity in the blood was still intact [^11^C]phenytoin. Metabolism appeared to be slower in the brain, or some metabolites might originate from the plasma with 83% intact [^11^C]phenytoin at 45 min, as shown in Table 4. The two radioactive nonpolar metabolites observed in the plasma were not detected in the brain. In addition, there was no difference in metabolism between tariquidar-treated and untreated rats.

**Table 4:** **Percentage of intact [**^**11**^**C]phenytoin at different time points after injection (*N*** **= 2)**

**Time (min)**	**Plasma (%)**	**Brain (%)**
5	60 ± 7	
10	50 ± 16	
15	38 ± 3	
25	28 ± 2	95 ± 1
30	32 ± 18	
45	19 ± 8	83 ± 5

Although moderate [^11^C]phenytoin metabolism in the periphery may be acceptable, it requires metabolite correction of plasma input functions for quantification of tracer kinetics, especially since it will be difficult to define a proper reference region.

### PET imaging

Typical summed ^11^C]phenytoin images (10 to 60 min) before and after tariquidar administration are shown in Figure 6. Cerebral uptake of ^11^C]phenytoin at baseline was low and rather homogenous throughout the brain. Time-activity curves, expressed in SUV, are shown in Figure 7. ^11^C]phenytoin displayed fast initial uptake followed by fairly fast clearance from the brain. After tariquidar administration, the brain SUV of ^11^C]phenytoin at 10 to 60 min increased on average by about 50%. The maximum SUV was around 0.8 and 0.9 for baseline and post-tariquidar scans, respectively. At 60 min, these values (corrected for metabolites) reduced to around 0.2 and 0.3 for baseline and post-tariquidar scans, respectively. More avid P-gp substrates, such as ^11^C]verapamil, have a brain SUV that is about half of these values at baseline [41].

**Figure 6:**
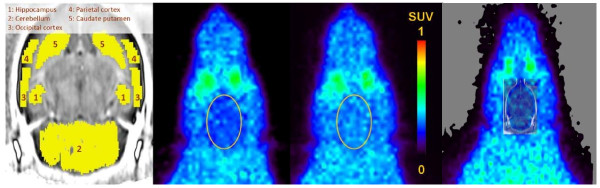
**Summed (10 to 60 min) images of [**^**11**^**C]phenytoin in the rat brain.** Before (second image) and 15 min after (third image) intravenous administration of tariquidar (15 mg·kg^−1^) in the rat. The brain is outlined in both images. Regions of interest used in the analysis are shown on an MR template image (left); the overlay image is shown on the right.

**Figure 7:**
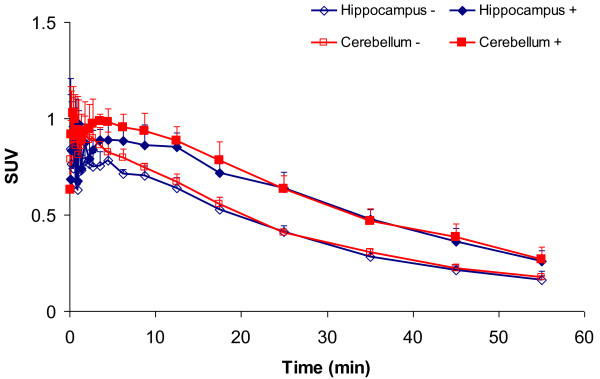
**[**^**11**^**C]phenytoin time-activity curves in selected rat brain regions.** Baseline (open symbols) and after administration of tariquidar (closed symbols). The injected dose was 17.1 ± 2.1 MBq. Error bars represent standard deviation.

Logan analysis showed that *V*_T_, i.e., the brain-to-plasma concentration ratio, of ^11^C]phenytoin increased by about 45% after administration of 15 mg·kg^−1^ tariquidar (Table 5), a dose that completely inhibits P-gp. The same dose resulted in a much larger increase in *V*_T_ (in the order of 1000%) for the established P-gp tracer *(R)*-^11^C]verapamil [42], indicating that ^11^C]phenytoin has a rather moderate affinity for P-gp.

**Table 5:** Results of Logan analysis using metabolite-corrected plasma input and metabolite-corrected brain data

	**Hippocampus**	**Occipital cortex**	**Parietal cortex**	**Caudate putamen**	**Cerebellum**
Baseline (*n* = 5) (*V*_T_)	0.96 ± 0.09	0.91 ± 0.06	0.91 ± 0.08	0.98 ± 0.08	1.02 ± 0.08
Tariquidar					
(*n* = 5) (*V*_T_)	1.43 ± 0.07	1.30 ± 0.11	1.35 ± 0.11	1.48 ± 0.12	1.48 ± 0.09
Probenecid					
(*n* = 2) (*V*_T_)	0.59 ± 0.00	0.59 ± 0.00	0.57 ± 0.03	0.57 ± 0.01	0.61 ± 0.01

The animals treated with probenecid experienced a severely depressed breathing frequency during 30 to 40 min after the probenecid administration, and this observation, i.e., discomfort to the animals, was the reason for not increasing the number of animals that were treated with probenecid. It is difficult to draw any conclusion from the studies with probenecid as the animals were in such poor conditions. Nevertheless, there was no indication of an increase in *V*_T_, which would have supported an interaction between [^11^C]phenytoin and MRP.

Initial [^11^C]phenytoin plasma concentrations after tariquidar treatment were somewhat lower than at baseline, but towards the end of the scans, plasma concentrations were similar. The initial decrease in plasma concentration after tariquidar administration is probably due to increased uptake of [^11^C]phenytoin in other tissues where P-gp is also inhibited.

Taken together, these initial preclinical studies showed that ^11^C]phenytoin has good tracer properties, i.e., moderate metabolic stability in the brain and favorable pharmacokinetics with a maximum brain concentration approximately 5 min after bolus injection in rats. In line with the initial hypothesis, ^11^C]phenytoin showed a higher baseline signal than more established tracers. Tariquidar predosing studies confirmed that ^11^C]phenytoin is indeed a weak P-gp substrate. It remains to be demonstrated, however, whether ^11^C]phenytoin will be a useful tracer to detect changes in P-gp expression and/or functionality. This can be studied in experimental animal models subjected to chemoconvulsants or electrical stimulation thereby inducing an epileptogenic process, which ultimately results in spontaneous epilepsy and pharmacoresistance [3,5,10]. Altered P-gp expression and functionality have been demonstrated in these models, and using ^11^C]phenytoin, this might be reflected in decreased uptake at baseline, altered response and/or tariquidar treatment or altered brain pharmacokinetics. It might even be easier to demonstrate in the (larger) human brain of pharmacoresistant epilepsy patients as altered P-gp functionality is likely to be local, allowing for a comparison of affected and unaffected cerebral regions.

## Conclusions

The radiosynthesis of [^11^C]phenytoin was improved by using [^11^C]CO rather than [^11^C]phosgene. [^11^C]phenytoin showed slow metabolism in the brain area *in vivo* but appeared to be a moderate P-gp substrate in rats. Compared with other P-gp substrate tracers, [^11^C]phenytoin displayed a higher baseline signal and a less pronounced increase after P-gp inhibition.

## Abbreviations

BBB: blood–brain barrier; DPPE: 1,2-bis(diphenylphosphino)ethane; DPPP: 1,3-bis(diphenylphosphino)propane; P-gp: P-glycoprotein; MRP: multi-resistant protein; PET: positron emission tomography; Rh(cod)_2_: Chloro(1,5-cyclooctadiene)rhodium(I) dimer; SUV: standardized uptake value; THF: tetrahydrofuran; *V*_T_: volume of distribution.

## Competing interests

The authors declare that they have no competing interests.

## Authors’ contributions

Each author contributed significantly to the submitted work, and all authors assisted in drafting the manuscript. JV, JE and ADW were in charge of the study, synthesis, radiosynthesis as well as acquiring, analyzing and interpreting the data. SS and ML were in charge of the animal study and analysis of the animal PET data. MPJM assisted with the radiosynthesis. MR was in charge of the metabolite analysis. ECML, RAV and AAL, with expertise in epilepsy research and preclinical study design and PET, contributed to the design of the study, analysis and interpretation of data. All authors read and approved the final manuscript.
